# Supernatants from culture of type I collagen-stimulated PBMC from patients with cutaneous systemic sclerosis versus localized scleroderma demonstrate suppression of MMP-1 by fibroblasts

**DOI:** 10.1007/s10067-012-1962-z

**Published:** 2012-02-25

**Authors:** Monica Brown, Arnold E. Postlethwaite, Linda K. Myers, Karen A. Hasty

**Affiliations:** 1 Le Bonheur Children’s Medical Center, Department of Pediatrics (Division of Clinical Immunology, Section of Rheumatology), University of Tennessee Health Science Center, 956 Court Avenue, Suite G326, Memphis, TN 38163 USA; 2 Department of Medicine (Division of Connective Tissue Diseases), University of Tennessee Health Science Center, Memphis, TN 38163 USA; 3 Department of Veterans Affairs Medical Center, Memphis, TN 38104 USA; 4 Department of Orthopedic Surgery, University of Tennessee Health Science Center, Memphis, TN 38163 USA; 5 Department of Pediatrics (Division of Clinical Immunology, Section of Rheumatology), Children’s Foundation Research Center at Le Bonheur Children’s Research Hospital, University of Tennessee Health Science Center, 956 Court Avenue, Suite G326, Memphis, TN 38163 USA

**Keywords:** IL-13, Diffuse, Localized scleroderma, MMP-1, PBMC, PDGF-BB, Scleroderma

## Abstract

Systemic sclerosis (SSc) is a chronic fibrosing disease characterized by vasculopathy, autoimmunity, and an accumulation of collagen in tissues. Numerous studies have shown that compared to healthy or diseased controls, the peripheral blood mononuclear cells (PBMC) from patients with SSc produce a variety of cytokines or proliferate when cultured with solubilized type I collagen (CI) or constituent α1(II) and α2(I) polypeptide chains. The purpose of this study was to determine whether PBMC isolated from patients with SSc and cultured in vitro with soluble CI elaborated soluble mediators that inhibit the production of collagenase (i.e., matrix metalloproteinase, MMP-1) by fibroblasts. Supernatants of CI-stimulated PBMC from juvenile and adult diffuse cutaneous (dc)SSc patients significantly reduced MMP-1 production by SSc dermal fibroblasts, while supernatants of CI-stimulated PBMC from patients with localized scleroderma (LS) did not. CI-stimulated PBMC culture supernatants from patients with dcSSc in contrast to patients with LS exhibited increased levels of platelet-derived growth factor (PDGF)-AA, PDGF-BB, TNF-α, IL-13, and EGF. Prolonged culture of SSc dermal fibroblasts with recombinant PDGF-BB or IL-13 inhibited the induction of MMP-1 in response to subsequent TNF-α stimulation. These data suggest that therapies aimed at reducing these cytokines may decrease collagen accumulation in SSc, preventing the development of chronic fibrosis.

## Introduction

Scleroderma “fibrosis of the skin” spectrum disorders include localized scleroderma (LS) variants and systemic sclerosis (SSc) the latter of which is further divided into diffuse cutaneous (dc)SSc, limited cutaneous (lc) SSc, and SSc sine scleroderma. The forms of SSc are differentiated primarily on the basis of the pattern of skin involvement, autoantibody association, and characteristics of internal organ involvement [1]. The main clinical differences between LS and SSc are the absence in the former of sclerodactyly, Raynaud's phenomenon, absence of widespread vasculopathy, nail fold capillary changes, and internal organ fibrosis. Five subtypes of LS or morphea are recognized by Peterson et al. and include “plaque,” “generalized,” “bullous,” “linear,” and “deep” [2]. The morphea classification of Laxer and Zulian [3] adds additional subgroups and recognizes “mixed variant morphea” which includes a combination of two or more subtypes in the same patient. Immunologic tests show that although autoantibodies are highly prevalent in both LS and SSc, they differ in prevalence and antigen specificity in these major divisions of scleroderma spectrum disease. For example, antinuclear antibodies are present in only 20% to 80% of patients with LS and in >90% of patients with SSc [4, 5]. Moreover, anti-topoisomerase II alpha antibody is present in up to 85% of patients with LS and in 14% of patients with SSc [4].

In this present study, we show profound differences in the immune responses of patients with dcSSC compared to patients with LS. Peripheral blood mononuclear cells (PBMC) from patients with dcSSc, but not patients with LS, cultured in vitro with soluble CI or α1(I) generate cytokine/growth factor-rich supernatants. These supernatants induce a reduction in matrix metalloproteinase (MMP)-1 production, when added to cultures of SSc dermal fibroblasts, but not to normal donor dermal fibroblast lines. We hypothesize that T cells from patients with dcSSc are activated by CI to produce cytokines that act on fibroblasts to reduce synthesis of MMP-1. A decrease in the skin fibroblast's ability to synthesize MMP-1 could shift the balance of collagen metabolism toward collagen excess resulting in fibrotic lesions.

In these studies, PBMC from patients with dcSSc or LS were cultured with or without CI, and the harvested supernatants were added to cultures of SSc fibroblasts to evaluate the effects on MMP-1 production. We found that long-term exposure to supernatants that had been generated by culturing CI-stimulated PBMC from adult or pediatric patients with dcSSc caused a significant decrease in the production of MMP-1 by SSc fibroblasts. In contrast, long-term exposure of SSc fibroblasts to supernatants from CI-stimulated PBMC from patients with LS did not induce suppression of MMP-1 production by tumor necrosis factor alpha (TNF-α)-stimulated dcSSc fibroblasts. These data suggest that therapies aimed at reducing the suppression of MMP may decrease collagen accumulation in skin and vital organs of patients with SSC, preventing the development of chronic fibrosis. Moreover, the results of this study may help elucidate the pathogenesis of impaired MMP-1 production by fibroblasts in SSc.

## Results

### Inhibition of MMP-1 production by dcSSc dermal fibroblasts chronically exposed in vitro to pooled culture supernatants from CI-stimulated PBMC from patients with dcSSc

To determine whether cytokine/growth factors present in supernatants from culture of CI-stimulated SSc PBMC would effect a change in MMP-1 production by dermal fibroblasts, we obtained PBMC from ten adult patients with dcSSc and stimulated them in vitro with CI or medium alone for 6 days. The harvested supernatants were pooled and added at 30% *v*/*v* to a culture of dermal fibroblasts from normal donors and from involved skin of patients with dcSSc for 14 days as described in the Patients and methods section, and MMP-1 production in response to TNF-α stimulation was assessed.

As shown in Table 1, culture of dcSSc fibroblasts for 14 days with supernatants from CI-stimulated adult dcSSc PBMC resulted in significant reduction in MMP-1 protein produced in response to TNF-α stimulation. Compared to normal fibroblasts, production of MMP-1 was also reduced in the dcSSc fibroblast cultures not stimulated with TNF-α but cultured for 14 days with CI-stimulated PBMC from patients with dcSSc. In contrast, fibroblasts from normal donors cultured for 2 weeks with the same pooled supernatants from CI-stimulated dcSSc PBMC and stimulated with TNF-α showed an actual enhancement of MMP-1 (Table 1).

**Table 1:** Effect of supernatant from type I collagen-stimulated PBMC (from patients with dcSSc) on MMP-1 production by dcSSc and normal fibroblasts

	Additions for 48 h	dcSSc fibroblast lines				Mean ± SE	Normal donor fibroblast lines				Mean ± SE	*P* value
Fibroblasts cultured with medium		004	008	1685	1822		1	3	2603	6858		
	PBS^a^	4	18	69	38	32 ± 14	5	15	60	29	27 ± 12	NS^b^
	TNF-α (5 ng/ml)	100	100	100	100	100	100	100	100	100	100	
Fibroblasts cultured with 30% *v*/*v* CI-stimulated dcSSc PBMC supernatant	PBS^a^	2	15	27	23	17 ± 5	125	69	92	22	77 ± 22	0.036^b^
	TNF-α (5 ng/ml)^a^	54	60	64	34	53 ± 7	578	413	210	106	327 ± 105	0.041^c^

MMP1 was measured by Western blot using pooled supernatants of CI-stimulated PBMC from ten dcSSc patients. The pooled PBMC supernatants were added at 30% *v*/*v* to cultures of normal and dsSSc fibroblasts for 14 days as described in the “Patients and methods” section. Normal and dcSSc fibroblasts cultured with pooled (*n* = 10) CI-stimulated dcSSc PBMC supernatants (bottom panel) were compared to the same cells cultured with the medium alone (top panel). The MMP-1 response to TNF-α in the medium alone was arbitrarily set at 100%

^a^MMP-1 calculated as a percent of that seen with TNF-α stimulation in fibroblasts cultured with the medium alone

^b^MMP-1 produced by SSc fibroblasts compared to normal dermal fibroblast cell lines following treatment with PBS

^c^MMP-1 produced by SSc fibroblasts compared to normal dermal fibroblast cell lines following treatment with TNF-α

Since the spectrum of scleroderma can range from very minimal involvement (LS) to extensive fibrosis (dcSSc), it was of interest to compare the effect of supernatants from the culture of PBMC from patients with LS to supernatants from the culture of PBMC from patients with dcSSc on MMP-1 production by SSc fibroblasts. In order to determine the variability among individual patients, we cultured PBMC with and without CI for 6 days and studied the effect of each individual donor PBMC supernatant on a single dcSSC dermal fibroblast line (SSc008) that became responsive (after subpassage 4) to MMP-1 upregulation by TNF-α.

**Fig. 1:**
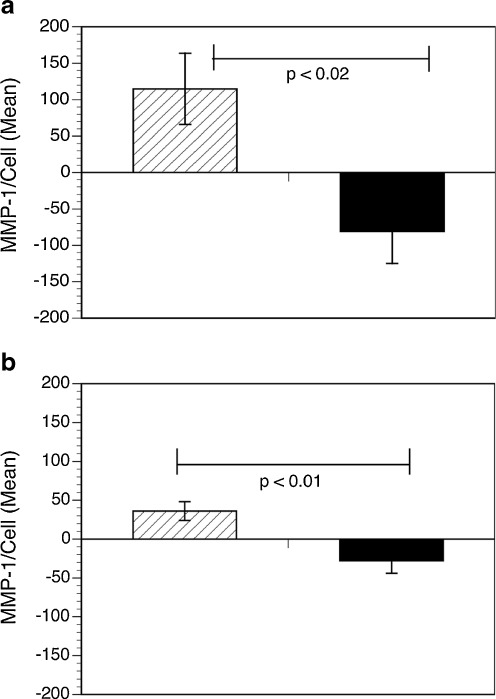
The effect of juvenile and adult dcSSc and LS PBMC supernatants on MMP-1 production by SSc fibroblasts. PBMC isolated from patients with dcSSc (*n* = 4; *solid bars*) and LS (*n* = 5; *hatched bars*) were stimulated with and without CI for 6 days, and the individual supernatants were tested for their effect on the MMP-1 production by SSc fibroblasts. Dermal fibroblasts from a patient with SSc (SSc008) were cultured for 21 days each with PBMC supernatants with (**a**) and without CI (**b**) after stimulation with TNF-α for 24 h. The amount of MMP-1 accumulating in the culture supernatants is shown measured by Western blot calculated as a percentage of MMP-1 from fibroblasts that were not co-cultured with PBMC supernatant, but were stimulated with TNF-α (control). Results were confirmed by ELISA (not shown). Results are expressed as mean ± SEM for MMP-1 content of the medium from the fibroblast cultures

PBMC from four dcSSc patients (one juvenile and three adults) and five juvenile patients with LS were cultured with or without native CI for 6 days. The harvested supernatants from the culture of PBMC from each of these patients were added at 30% *v*/*v* to cultures of dermal fibroblasts (SSc008) for 21 days with fresh media and PBMC supernatant added every 3 days. After 21 days, TNF-α was added to the cultures of SSc008 fibroblasts, to stimulate production of MMP-1. As illustrated in Fig. 1, the SSc 008 fibroblast line produced significantly less MMP-1 after being cultured for 21 days with CI-stimulated supernatants from dcSSc patients compared to fibroblasts cultured with CI-stimulated supernatants from LS patients.

### Cytokine profile for CI-stimulated PBMC supernatants from juvenile and adult dcSSc

**Fig. 2:**
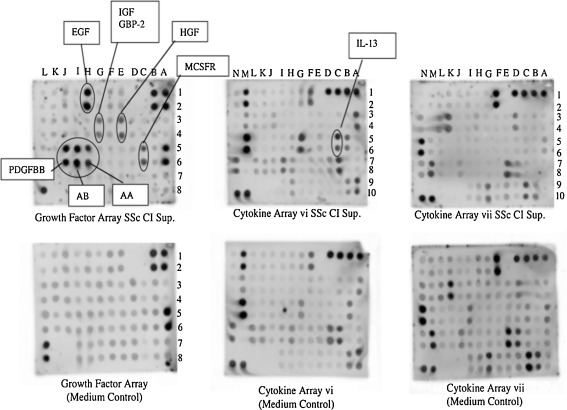
RayBio Human Cytokine Antibody Array of pooled culture supernatants from PBMC from ten adult patients with dcSSc stimulated for 6 days with 10 μg/ml native bovine CI. After 6 days of incubation of PBMC plus 10 μg/ml CI, culture supernatants were pooled and analyzed, along with the culture medium alone (complete DMEM) as a control, in a RayBio Cytokine and Growth Factor Antibody Array assay. The assays used contained antibodies to 120 cytokines, and receptors and 41 growth factors impregnated in small circles on membranes along with positive and negative controls. All antibodies are bound in small duplicate circles on the membranes. *Drawn circles* indicate cytokines that were enhanced above the background. Unstimulated PBMC produce small amounts of cytokines spontaneously thereby preventing their use as controls

**Fig. 3:**
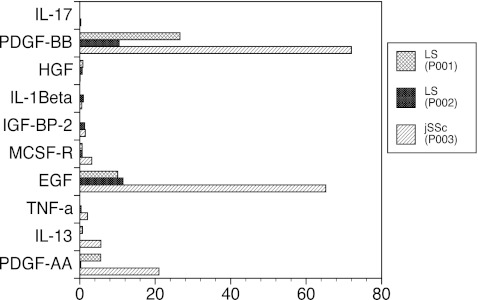
Cytokine profile supernatants from juvenile dcSSc and LS PBMC stimulated with CI. PBMC from one patient with dcSSc (P003) and two patients with LS (P002 and P001) were cultured individually for 6 days with CI, and the supernatants were collected and analyzed to be tested using RayBio Cytokine and Growth Factor Antibody Array assay. The data points represent individual patients. The patient with dcSSC had increased PDGF-AA, PDGF-BB, IL-13, TNF-α, and EGF as compared to PBMC from LS patients (P002 and P001). Comparisons are made to the positive endogenous control provided by the manufacturer

The inhibition of MMP-1 described above is very likely induced by cytokines or growth factors secreted by peripheral blood cells. In order to determine the cytokine profile of supernatants from culture of CI-stimulated dcSSc PBMC, we pooled CI-stimulated PBMC culture supernatants from ten adult patients with dcSSc and incubated these supernatants on RayBioplex membrane arrays to test for presence of individual cytokines/growth factors. The pooled supernatants were tested on three different cytokine or growth factor arrays and were found to be abundant in platelet-derived growth factor (PDGF)-AA, PDGF-BB, PDGF-AB, epidermal growth factor (EGF), insulin-like growth factor binding protein 2 (IGFBP-2), hepatocyte growth factor (HGF), macrophage colony stimulating factor receptor (MCSFR), and interleukin-13 (IL-13) when compared with the culture medium alone (Fig. 2). We further evaluated the cytokine profile of supernatants from CI-stimulated PBMC from one pediatric dcSSC patient (Fig. 3, P003) and two pediatric LS patients (Fig. 3, P001, P002). We cultured PBMC with CI for 6 days from these patients, harvested the supernatant, and incubated these supernatants on RayBioplex membrane arrays as per manufacturer's instructions. As shown in Fig. 3, the cytokine profile of supernatants from CI-stimulated PBMC from the juvenile dcSSc patient revealed an increase in PDGF-AA, PDGF-BB, IL-13, TNF-α, and EGF when compared to the supernatant from CI-stimulated PBMC from patients with LS. Although the numbers are small, the PBMC from the dcSSC patient clearly secreted greater amounts of PDGF-AA, PDGF-BB, IL-13, TNF-α, and EGF when compared to supernatants collected from the culture of CI-stimulated PBMC from LS patients. These results compare favorably to the cytokine profile in supernatants from the culture of CI-stimulated PBMC from adult patients with dcSSc (Figs. 2 and 3).

### Effects of long-term incubation of SSc dermal fibroblast with profibrotic cytokines

**Fig. 4:**
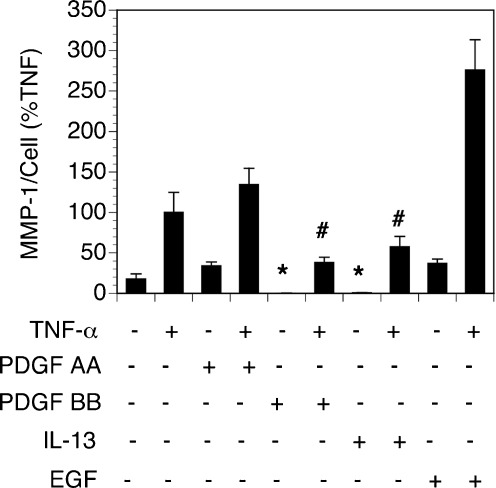
SSc dermal fibroblasts cultured for 14 days with pro-inflammatory cytokines followed by stimulation with TNF-α for 24 h. Inhibition of constitutively produced MMP-1 as well as TNF-α-stimulated MMP-1 production was observed when SSc 008 fibroblasts were cultured with PDGF-BB and IL-13 when compared to SSc fibroblasts cultured with the medium alone for 14 days ± TNF-α. MMP-1 was increased when fibroblasts were cultured for 14 days with PDGF-AA or EGF and then stimulated 24 h with TNF-α. *P < 0.05 when compared to supernatants from fibroblasts cultured with the complete medium alone (*first bar* on the left). ^#^ *P* < 0.05 when compared to supernatants from fibroblasts cultured with the complete medium plus TNF (*second bar* from the left)

In a separate experiment, PDGF-AA, PDGF-BB, IL-13, and EGF were added for 14 days individually at varying doses to cultures of SSc fibroblasts to assess their effect on MMP-1 production induced by TNF-α. Although multiple concentrations were tested, the data shown in Fig. 4 illustrate the most effective dose for each cytokine. Long-term exposure of SSc 008 fibroblasts to PDGF-BB and IL-13 significantly inhibited MMP-1 production when the fibroblasts were subsequently stimulated with TNF-α. These data suggest that each of these cytokines may be responsible for the inhibitory effect on fibroblast MMP-1 production induced by supernatants from CI-stimulated PBMC from patients with dcSSc. In contrast, long-term exposure of SSc 008 fibroblasts to PDGF-AA and EGF resulted in enhanced TNF-α stimulation of MMP-1 production (Fig. 4). Although EGF is known to upregulate TGF-β via PI 3-kinase/Akt signaling pathway in dermal fibroblasts [6], the upregulation of TGF-β by EGF in the present experiments would not impact MMP-1 production, as activation of latent TGF-β does not occur spontaneously in culture, and active TGF-β suppresses MMP-1 production by fibroblasts [7].

## Discussion

Our results show that soluble mediators from PBMC from patients with dcSSc inhibit collagenase expression in cultured SSc dermal fibroblasts. Supernatants of soluble CI-stimulated PBMC from dcSSc patients exerted profound suppression of production of MMP-1 by SSc fibroblasts. Pooled supernatants from a group of adult dcSSc patients and individual supernatants from juvenile and adult dcSSc patients significantly reduced the expression of MMP-1 by SSc fibroblasts. Suppression of constitutive production as well as TNF-α-stimulated production of MMP-1 by SSc fibroblasts was observed when these fibroblasts were chronically exposed to supernatants from CI-stimulated dcSSc PBMC. In contrast, prolonged culture of SSc fibroblasts with supernatants of CI-stimulated PBMC from patients with LS did not induce the MMP-1 suppressive phenotype in SSc fibroblasts.

TNF-α inhibits collagen synthesis and increases synthesis of MMP-1 when added to cultures of normal human fibroblasts [8]. Unstimulated PBMC from patients with lc- or dcSSc in culture produce greater amounts of TNF-α than PBMC cultured from healthy volunteers, and the most TNF-α was produced by unstimulated PBMC from patients with early (<3-year duration) dcSSc [9]. Higher amounts of TNF-α are also expressed in the skin of patients with SSc [10]. Although TNF-α is abundantly produced in SSc patients, our data in this report suggest that cytokines produced by CI-activated PBMC from patients with dcSSc may render fibroblasts insensitive to TNF-α upregulation of MMP-1 and thereby contribute to dermal fibrosis.

The pathogenesis of fibrosis in LS and SSc is still unresolved, but a role for autoimmunity in effecting fibrosis is suggested by early tissue infiltrates of mononuclear cells that have the capability of producing an array of cytokines and growth factors that are known to mediate fibrogenesis. The histologic features of the lesional skin of patients with LS and SSc are largely indistinguishable and are characterized in the early stages by perivascular infiltration of predominantly mature lymphocytes, T lymphoblasts, immature plasma cells, mature plasma cells, fibroblasts, fibrocytes, fibroblast-like cells, macrophages, undifferentiated mesenchymal cells, and monocytes [11]. Later, additional reports have described the cellular infiltrates more precisely. Roumm et al. analyzed the infiltrates in the lesional skin of patients with SSc using monoclonal antibodies to cell surface markers and found the mononuclear cell (MNC) infiltrates were composed predominately of activated CD4^+^ and CD8^+^ T cells in a ratio of 2.4 to 1, and few B cells and monocytes were observed [12]. Importantly, in this larger cohort study of 115 patients with SSc, significant correlations were observed between the degree of MNC infiltration and both the degree and progression of skin thickening [12].

Separate studies have shown that when PBMC from patients with lc or dcSSc (in contrast to healthy volunteers) are cultured with soluble CI or constituent α1(I) or α2(I) chains, there is increased production of cytokines including IL-2, IL-6, IFN-γ, IL-10; increased production of monocyte chemoattractants, and increased T cell proliferation [13–18]. In addition, Warrington et al. labeled PBMC from patients with lcSSc, dcSSc, and rheumatoid arthritis and healthy volunteers with carboxyfluorescein diacetate succinimidyl ester (CFSE) and cultured these CFSE-labeled PBMC for 14 days with and without soluble native bovine CI and β1, two chains [dimers formed by α1(I) and α2(I)] [19]. Analysis by flow cytometry showed a T cell proliferative response to these CI α chains occurring in 32% of SSc patients but only in 3.6% healthy volunteers (1 of 19 volunteers) and in 0% RA patients (0 of 9) [19]. The proliferating T cells expressed CD4^+^, activated (CD25^+^) memory (CD45 RO^+^) phenotype [19]. The T cell lines generated by prolonged culture (20–30 days culture) of sorted CI-reactive T cells with CI, irradiated autologous PBMC, and IL-2 were analyzed after resting for 10 days and then stimulated for 72 h with plate-bound anti-CD3 and anti CD28. These T cell lines then produced abundant Th1 cytokines, IFN-γ, IL-2, TNF-α, lower amounts of Th2 cytokines (IL-4, IL-6, and IL-10), and abundant chemotactic chemokine IL-8 [19].

While this study does not establish whether the diminished production of MMP-1 that we have observed is due to T cell autoimmunity to CI or due to a costimulatory effect of CI on activated T cells, we favor an antigenic role of CI in triggering production of cytokines. We have previously shown that CI-specific T cell clones can be grown from peripheral blood of SSc patients [19]. Moreover, the fact that soluble CI was used rather than plate-bound CI in the PBMC culture favors the concept that CI functions as an antigen, which triggers the production of cytokines which cause SSc fibroblasts to assume a phenotype of impaired MMP-1 production.

The pathogenesis of SSc is marked by the overabundance of collagen and skin fibrosis where collagen fibers accumulate in the dermis. Uncontrolled production of collagen and other extracellular matrix proteins produced by fibroblasts contributes to the fibrosis [20]. MMP-1 is the major proteinase involved in the degradation of CI and CIII. Although decreases in MMP-1 in fibroblasts from patients with early SSc have been reported [21], the results have been controversial [22]. Our data support the concept that an important component of the pathogenesis of SSc is the diminished capacity for production of MMP-1 by fibroblasts. Our data are unique in indicating that the reduction of fibroblast MMP-1 is mediated by cytokines derived from PBMC stimulated by CI. It is possible that CI is functioning as an autoantigen or a co-stimulator of T cells in eliciting these cytokines.

The expression of IL-13 and its receptor complex is increased in SSc skin samples, correlating with modified Rodnan skin scores (Aliprantis; ACR 2010) and with nail fold capillary patterns [23–25]. Our studies demonstrate that pooled supernatants from adult SSc PBMC co-cultured with CI contain significant levels of many profibrotic cytokines compared to controls (PDGF-AA, PDGF-BB, PDGF-AB, EGF, IGFBP-2, HGF, MCSFR, and IL-13) [6, 26–29]. Our studies of pediatric patients with SSc have shown that CI induces increased expression of PDGF-AA, PDGF-BB, EGF TNF-α, and IL-13. The overlap of cytokines found in juvenile and adult SSc compared to that seen in localized disease may in fact be reflective of severity of skin and organ involvement between these two diseases. Moreover, the functional importance of these cytokines is demonstrated by our results that prolonged incubation of PDGF-BB and IL-13 significantly inhibited TNF-α, induced MMP-1 production by SSc dermal fibroblasts, and highlight the importance of these cytokines in dcSSc. Together, the interaction of PBMC with CI and the elicited cytokines stimulates a pathway inherent in SSc fibroblasts that inhibits MMP-1 expression and contributes to the fibrosis in this disease.

In the present study, we have shown that cytokines produced by CI-stimulated PBMC from patients with dcSSc suppress MMP-1 production. These data suggest a role for activation of lymphocytes and/or monocytes in a subgroup of patients with dcSSc and suggest a cytokine-mediated pathway involved in the pathogenesis of the extensive fibrosis seen in these patients. Since we have shown that exposure to oral CI may benefit late-phase dcSSc, our new results have important implications for future treatments for SSc [30].

## Patients and methods

### Patient population

This study was conducted using protocols approved by the Institutional Review Board of participating institutions. The Declaration of Helsinki protocols were followed; patients gave their written informed consent. Juvenile patients with dcSSc (ages 16 years or less) were included in this study who met the PREs/ACR/EULAR classification of juvenile systemic sclerosis [31]. Adult patients (aged 18–70) met the American College of Rheumatology preliminary criteria for diagnosis of SSc. Patients with LS met the classification criteria for morphea or linear scleroderma [2, 32].

### Preparation of bovine native CI and α1(I) chains

Native CI was isolated and purified from fetal bovine skin as previously described [33]. Homogeneity of CI and α1(I) was confirmed using SDS-PAGE and by cyanogen bromide peptide mapping. The purified CI was dissolved in cold 0.1 M acetic acid at 4 mg/ml and stored frozen at −70°C. α1(I) was isolated from denatured bovine CI as previously described [34]. Purified α1(I) was lyophilized and stored at −20°C in a desiccator.

### Specific reagents

Human recombinant TNF-α, IL-13, PDGF-AA, PDGF-BB, and EGF were purchased from R&D systems (Minneapolis, MN). Anti-rabbit IgG was purchased from GE Healthcare (UK). The antibody to human MMP-1 (rabbit) was made to a latent and truncated peptide, expressed in *Escherichia coli*, and purified in the laboratory. MMP-1 was detected by ELISA (R&D systems) and cytokines by RayBio Human Cytokine Antibody Arrays (RayBiotech, Inc.).

### Production of PBMC culture supernatants

Blood samples were collected in EGTA-containing glass tubes and the PBMC isolated over Histopaque (Sigma Chemical Co., St. Louis, MO) cushions by isopynic centrifugation. The PBMC were plated at 2 × 10^6^ cells per well, cultured in Dulbeccos's high-glucose modified eagle medium (DMEM) containing 10% fetal bovine serum (FBS), penicillin (100 units/ml), streptomycin (100 ug/ml), and 100× Glutamax (Invitrogen Corp.) [19] (hereafter called “Complete DMEM”). Cells were cultured at 37°C with 5% CO_2_ for 6 days with and without purified bovine collagen α1(I) (25 μg/ml) after which the supernatants were collected, pooled, and stored in −80°C until use. In one experiment, PBMC from ten adult patients with dcSSc were collected and cultured for 6 days with native bovine CI (10 μg/ml), prior to pooling and storing at −70°C.

### Dermal fibroblast cultures

Four normal and four SSc fibroblast lines (two from biopsies of lesional and two from non-lesional skin of dsSSc patients) were grown for 2–3 weeks in eagle minimum essential medium, with 9% fetal calf serum (FCS), 100 μ/ml penicillin, 100 μg/ml streptomycin, and amphotericin B (1 μg/ml) hereafter referred to as “complete medium.” Thirty percent (by volume) of supernatant from a culture of CI-stimulated dcSSc PBMC (from a pool of ten adult patients with dcSSc, sterile filtered and aliquoted into individual vials) or 30% medium (by volume) alone or plus native CI (10 μg/ml) of high-glucose DMEM containing the same lot of FBS used in the SSc PBMC cultures was added to each plate containing each cell line. The medium in the cultures was changed every 7 days, and a new SSc PBMC culture supernatant or medium control (each 30% by volume) was added at each refeeding. Cells were then trypsinized and passed into new 100-mm tissue culture plates and cultured for 3 days in the complete medium. Fibroblasts were then trypsinized and transferred to Costar tissue culture plates at a density of 10^5^ cells/well and grown for 3 days in the complete medium. The medium was then changed to the complete medium containing 5% FBS for 24 h, changed again to a fresh complete medium with 5% FCS containing either 5 ng/ml human recombinant TNF-α (R and D Systems, Minneapolis, MN) in PBS containing 0.1% bovine serum albumin (BSA, Sigma) or PBS with 0.1% BSA as a control. After 48 h, culture supernatants were harvested. Culture supernatants were assayed by ELISA or Western blot analysis to quantitate MMP-1 protein as previously described [35].

### Culture of SSc fibroblasts with supernatants from PBMC cultures

Fibroblasts were grown from dermal biopsies from similar locations on the forearm of normal volunteers or patients with dcSSc. SSc fibroblast cell line 008 (SSc 008) passages 5–15, that had regained the ability to synthesize MMP-1 upon stimulation with TNF-α, was also used for comparing the effect of the supernatant from the culture of PBMC from different donors. This SSc fibroblast line, grown from the fibrotic skin on the volar forearm of a patient with dcSSc, was resistant to TNF-α stimulation of MMP-1 synthesis during the first few subpassages but became more responsive to TNF-α after passage. The SSc 008 fibroblasts were plated at 5 × 10^4^ cells/well in Costar 24 well and grown in high-glucose DMEM containing 10% FCS. Supernatants from 6-day PBMC cultures were removed from −80°C storage, allowed to thaw at 37°C, and then centrifuged at 190 relative centrifugal force for 10 min. Additions to fibroblast cultures consisted of the following: collagen alone in complete DMEM (complete DMEM incubated 6 days with 12.5 μl (12.5 μg) of αl(I) without PBMC in wells), PBMC supernatant (PBMC; DMEM incubated 6 days with PBMC from scleroderma patients), and collagen supernatant (+CI/PBMC; DMEM incubated for 6 days with PBMC and 12.5 μl of 12.5 μg α1(I)). The PBMC supernatants or complete medium was added at 30% *v*/*v* to triplicate well cultures of SSc 08 fibroblasts. The medium was changed every 3 days for a total of 21 days, then DMEM supplemented with 5% FBS was placed on the fibroblasts for 24 h prior to adding TNF-α 5 ng/ml for an additional 24 h. Fibroblast cultures were then collected and stored at 4°C until analysis by Western blot or ELISA to quantitate MMP-1 level. Fibroblasts were harvested for cell number quantification using CyQuant (Invitrogen, Eugene, OR).

### Analysis of MMP-1

Fibroblast culture supernatants were analyzed for MMP-1 protein by Western blot analysis or by ELISA. Supernatants were resolved on 9% SDS-PAGE and electrotransferred to a PVDF membrane, blocked in 5% powdered milk in TBS (1 M Tris, pH 7.4; 4 M NaCl; deionized water) for 1 h, then soaked in primary MMP-1 antibody. The membrane was washed and incubated with anti-MMP-1 antibody, and then, alkaline phosphatase-conjugated anti-rabbit IgG was used to visualize immunoreactive bands with ECF substrate. Densities were normalized to cell proliferation values (number of cells) and calculated as a percent of the TNF-α control value (Storm 860 Molecular Dynamics, Sunnyvale, CA, and Typhoon GE Healthcare Biosciences AB, Sweden) and analyzed using ImageQuant TL 7.0 (GE Healthcare, Sweden). In some experiments, human total MMP-1 Duoset (R&D) was used to quantify MMP-1.

### Cytokine analysis

Cytokine analysis was performed on 6-day complete DMEM and +CI/PBMC pooled supernatants obtained from 6-day culture of PBMC from ten dcSSc patients cultured with 10 μg/ml native CI using RayBio Human Cytokine Antibody Arrays VI, 6.1, and VI, 7.1 (RayBiotech, Inc.).

Further analysis was done on CI-stimulated 6-day PBMC supernatants from two juvenile LS patients and one dsSSc juvenile patient. For this analysis, membrane arrays were custom made for by the manufacturer for detection of PDGF-AA, PDGF-BB, IL-13, TNF-α, EGF, MCSCR, insulin-like binding protein 2 (IGFBP-2), IL-1β, HGF, and IL-17. These cytokines were selected based on the preliminary cytokine data obtained from pooled supernatants from dcSSc patients. All procedures for this part of the experiment were followed according to the manufacturer's instructions. Each blot of cytokine array membranes was normalized to irrelevant endogenous controls included on the blot by the manufacturer to allow comparisons between blots and analyzed using Typhoon chemiluminescence.

To study the effect of long-term exposure of fibroblasts to cytokines/growth factors, fibroblasts were plated in 100-mm tissue culture plates at 4 × 10^5^ cells per plate and then cultured with human recombinant PDGF-AA, PDGF-BB, IL-13, and EGF for a total of 14 days. The medium was changed every 72 h with addition of fresh medium containing the specific cytokine/growth factor. Fibroblasts were passed by trysinization and then plated with the complete medium containing 10% FCS in Costar 24-well tissue culture plates at 5 × 10^4^ per well. These fibroblasts were then grown in the complete medium for 1 week with medium change every 72 h. Fibroblasts were then grown in DMEM containing 5% FBS for 72 h, followed by 24 h with TNF-α stimulation. Human total MMP-1 Duoset was used to determine MMP-1.

### Statistical analysis

An all-or-none comparison was made between groups. ANOVA and Fisher's exact test were used for statistical analysis due to a small sample size. *P* < 0.05 was considered significant. Data are reported with bars indicating the standard deviation of the mean.
